# Adipose tissue plasticity and the pleiotropic roles of BMP signaling

**DOI:** 10.1016/j.jbc.2021.100678

**Published:** 2021-04-17

**Authors:** Shuwen Qian, Yan Tang, Qi-Qun Tang

**Affiliations:** The Key Laboratory of Metabolism and Molecular Medicine of the Ministry of Education, Department of Biochemistry and Molecular Biology of School of Basic Medical Sciences, and Department of Endocrinology and Metabolism of Zhongshan Hospital, Fudan University, Shanghai, China

**Keywords:** BMPs, adipogenesis, plasticity, adipocyte, beige, macrophage, ATM, adipose tissue macrophage, BAT, brown adipose tissue, BMP, bone morphogenetic protein, GDF, growth/differentiation factor, HALS, highly active antiretroviral treatment–associated lipodystrophy syndrome, IL, interleukin, PDGFRβ, platelet-derived growth factor receptor beta, PPARγ, proliferator-activated receptor gamma, TGF, transforming growth factor, TNFα, tumor necrosis factor α, UCP1, uncoupling protein 1, WAT, white adipose tissue, Zfp423, zinc-finger protein 423

## Abstract

Adipose tissues, including white, beige, and brown adipose tissue, have evolved to be highly dynamic organs. Adipose tissues undergo profound changes during development and regeneration and readily undergo remodeling to meet the demands of an everchanging metabolic landscape. The dynamics are determined by the high plasticity of adipose tissues, which contain various cell types: adipocytes, immune cells, endothelial cells, nerves, and fibroblasts. There are numerous proteins that participate in regulating the plasticity of adipose tissues. Among these, bone morphogenetic proteins (BMPs) were initially found to regulate the differentiation of adipocytes, and they are being reported to have pleiotropic functions by emerging studies. Here, in the first half of the article, we summarize the plasticity of adipocytes and macrophages, which are two groups of cells targeted by BMP signaling in adipose tissues. We then review how BMPs regulate the differentiation, death, and lipid metabolism of adipocytes. In addition, the potential role of BMPs in regulating adipose tissue macrophages is considered. Finally, the expression of BMPs in adipose tissues and their metabolic relevance are discussed.

Adipose tissues are highly dynamic organs, as illustrated by the rapid changes that occur upon acute nutritional stresses (1, 2), as well as the continued remodeling associated with chronic stimuli or aging (3, 4, 5, 6). As a result, adipose tissues either maintain the balance between multiple biological processes or enter a pathologic state of either obesity (fat gain) or lipodystrophy (fat loss). Both the expansion and loss of adipose tissues involve the coordinated responses of multiple cell types: adipocytes, immune cells, endothelial cells, nerves, and fibroblasts (7). This coordination is reflected by the communication between different types of cells and a balance between activity extremes, for example, cell growth and death, lipogenesis and lipolysis, and proinflammatory and antiinflammatory activity. These cellular and molecular features affect the plasticity of adipose tissues.

The plasticity of adipocyte tissues determines how adipose tissues accommodate to environmental cues. Responding to changes in factors such as food availability and environmental temperature, exercise and the immune response to infection all require energy. There are different types of adipose tissues for these purposes. White adipose tissue (WAT) stores energy in the form of triglycerides after consumption of a nutrient load, and it also releases fatty acids through quick mobilization to ensure that peripheral energy demands are met. In contrast, brown adipose tissue (BAT) dissipates energy *via* nonshivering thermogenesis (8). The thermogenic capacity of BAT is because of its high numbers of mitochondria and high levels of uncoupling protein 1 (UCP1), which uncouples oxidative phosphorylation to generate heat instead of ATP (9, 10, 11, 12). Classical BAT concentrates in the area between the two scapula (interscapular) on the back but disappears in adulthood. Intriguingly, over the past 2 decades, research has revealed a third type of adipose tissue, termed beige/brite/brown-like adipose tissue (13, 14, 15, 16, 17, 18, 19). Beige adipocytes are aroused in white fat depots and respond to stimuli such as cold and exercise-induced hormones (13, 15, 19, 20, 21, 22, 23, 24). As beige adipocytes contain abundant mitochondria and are active in dissipating energy, they have drawn much attention as a therapeutic target for obesity.

For decades, studies have identified numerous factors that participate in regulating adipose biology, among which bone morphogenetic proteins (BMPs) have been relatively newly discovered. The novelty of BMPs might be because the majority of studies on BMPs have focused on developmental processes such as the determination of the body axis, germ layer specification, organ development, tissue morphogenesis, and cell fate specification (25, 26, 27, 28). Emerging studies are demonstrating the important and pleiotropic roles of BMPs and their signaling in adipose regulation. An understanding of the pleiotropic functions of BMPs may identify BMP actions in adipose tissues as therapeutic targets for metabolic disorders.

## Origin of adipocytes

Adipocytes, the parenchymal component of adipose tissues, are known to have mesodermal or neuroectodermal origins (29, 30, 31, 32). Human adipose tissues first appear and progressively develop from the 14th to 24th week of gestation (33). In mice, lipid staining with BODIPY shows that subcutaneous, retroperitoneal (behind the peritoneum in the abdomen), and brown adipose tissue adipocytes already contain lipid components on postnatal day 1 (P1). By contrast, BODIPY+ adipocytes in epididymal adipose tissue start to appear on P7 (34). Later on, an AdipoChaser mouse that features mature adipocytes labeled by adiponectin promoter-driven LacZ expression has been developed. The model is doxycycline inducible, thus enabling temporally controlled identification of newly formed adipocytes. It has been used to reveal that adipocytes at subcutaneous depots begin to form at embryonic days 14 to 18 (E14–E18) (35).

**Figure 1:**
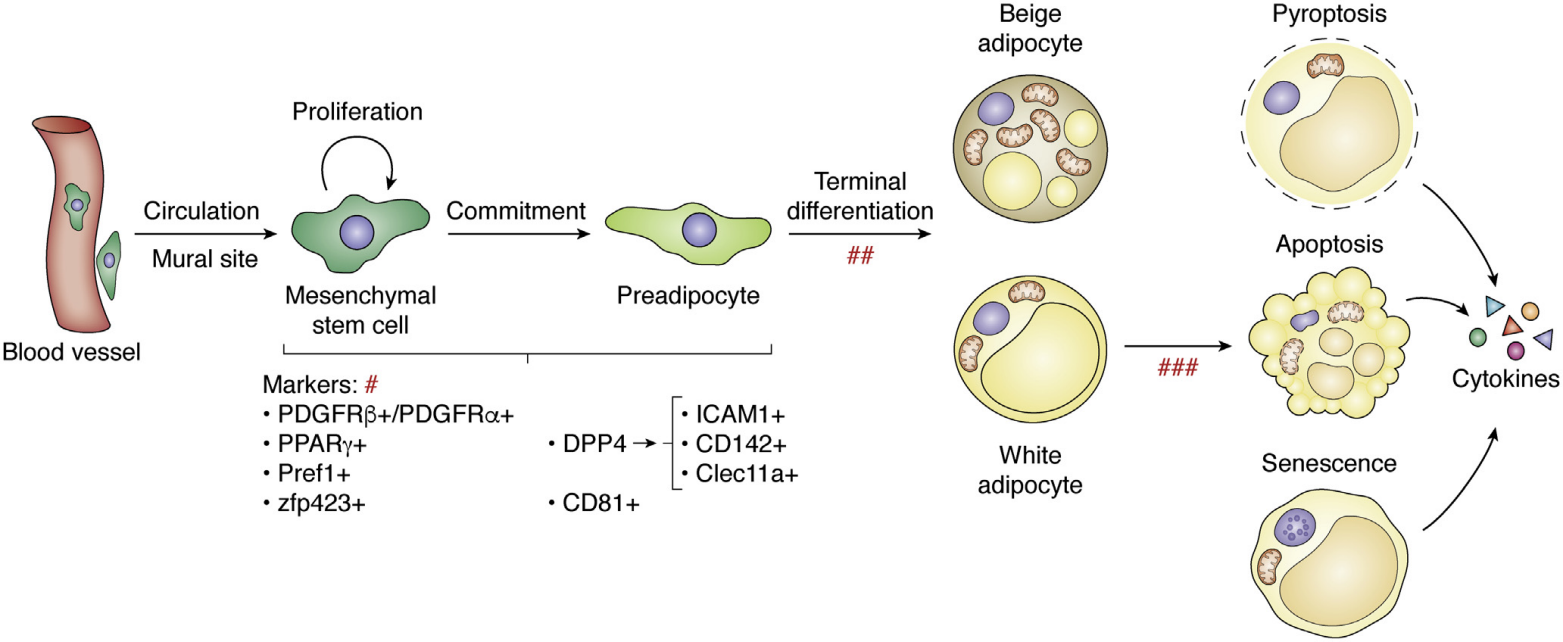
**Plasticity of adipocytes.** Adipocyte development is defined as commitment and terminal differentiation. Mesenchymal stem cells are multipotent. They are either resident (probable endothelial origin) or recruited from circulation. Some markers identify stem cells to be multipotent cells, and other committed preadipocytes. These markers may overlap, and their developmental timing and interrelationship are not clear (#). Beige and white adipocytes derive from distinguished precursors (##). Adipocyte death can be pyroptosis, apoptosis, and senescence (exemplified white adipocytes), which are initiated by different stresses, and eventually may coexistent in 1 cell (###). The dead or senescent cells are accompanied with increased release of inflammatory cytokines. PDGFRβ, platelet-derived growth factor receptor beta; PPARγ, proliferator-activated receptor gamma; Zfp423, zinc-finger protein 423.

Adipocyte development is generally regarded to contain two stages, the transition from multipotent stem cells to lineage-specific preadipocytes (termed commitment) and the transition from preadipocytes to fully developed adipocytes (termed terminal differentiation) (Fig. 1). Although the timing of the appearance of lipid-containing adipocytes has been identified, the exact timing of the beginning of adipocyte development is hard to determine because the characteristics that define adipocyte precursors are still under debate. Histological studies show that the blood vessel structures have formed before adipocyte development in some anatomic locations of the fetus (36). Vasculature formation also occurs before the development of epididymal adipose tissue, which occurs postnatally (34). This observation supports the hypothesis that adipocytes originate from a subset of blood vessels. Several groups have demonstrated that the mural cells of blood microvessels are multipotent and implicated in the development of tissues, including adipose tissues (37, 38, 39, 40, 41). Mural cells bear the markers platelet-derived growth factor receptor beta (PDGFRβ), alpha-smooth muscle actin, and NG2 (37). Studies carried out by Gupta R’s group tracked the adipogenic fate of PDGFRβ-positive cells *via* genetic marking and showed that adipose mural cells (PDGFRβ+) contribute to the increased numbers of adipocytes (hyperplasia) seen in both obese and cold-exposure contexts (42). The development of adipocytes from PDGFRβ+ cells for healthy expansion of visceral fat has a protective effect against metabolic impairment (43). Several other studies have identified PDGFRα as an adipocyte precursor marker (44, 45, 46, 47), with PDGFRα+ cells giving rise to either brown adipocytes or white adipocytes. Most recently, by using mosaic lineage labeling, Sun *et al.* showed that adipocytes are derived from the PDGFRα+ lineage during postnatal growth and adulthood. In contrast, adipocytes are only derived from the mosaic PDGFRβ+ lineage during postnatal growth (48). One study that labeled cells with peroxisome proliferator-activated receptor gamma (PPARγ)-driven GFP identified PPARγ as a marker of adipocyte precursors (40). Of note, PPARγ-GFP+ cells were localized within a subset of cells of the mural compartment in the vasculature. Similarly, zinc-finger protein 423 (Zfp423) has been identified as an essential determinant of preadipocytes because Zfp423-expressing cells can undergo robust adipogenesis (49). It is interesting that Zfp423 expression is also found in some mural cells. Although both PPARγ and Zfp423 are designated markers of adipocyte precursors, there are still not enough data to clearly depict the chronological order of the appearance of PPARγ and Zfp423 expression during the commitment stage or the specific relationships between PPARγ and Zfp423. Notably, a very small subset of CD31+ (a commonly used marker for endothelial cells) capillary endothelial cells exhibit clear Zfp423 expression (49). This result may be evidence for the hypothesis that adipocytes are of endothelial origin, which is also supported by lineage tracing experiments using the VE-cadherin (an endothelial cell-specific adhesion molecule) promoter (50). A VE-cadherin construct including LacZ showed expression that was localized within endothelial cells, as well as preadipocytes and adipocytes at both white and brown fat depots, suggesting that endothelial cells give rise to white and brown adipocytes (50). Most recently, with advances in single-cell sequencing technology, more molecules, such as DPP4, ICAM1, CD142, and Clec11a, have been defined as adipocyte progenitor markers, with DPP4 preceding ICAM1, CD142, and Clec11a (51, 52, 53). Single-cell sequencing has also identified CD81 as a marker of preadipocytes, more specifically, of beige adipocytes [49]. All these reported markers may overlap, and many of their developmental interrelationships are not yet clear (Fig. 1).

In addition to the cells resident in blood vessels, mesenchymal stem cells from circulation contribute to a portion of the adipocytes that exist in various fat depots (54). The origin of the cells used to generate newly formed adipocytes may depend on stimuli. Thiazolidinedione induces the recruitment of adipocytes from circulation, and cold seems to recruit adipocytes from both resident site and circulation (4, 42, 54, 55, 56, 57, 58). Adipocytes derived from different cell reservoirs exhibit heterogeneous characteristics and gain adipose tissues flexibility for adaptation. As mentioned earlier, from a developmental point of view, adipocytes are of mesoderm or neuroectoderm in origin. Lineage-tracing studies have shown that both brown adipocytes and myocytes are derived from Myf5+ and PAX7+ progenitors that originate in the paraxial mesoderm (59, 60, 61, 62). It was previously thought that white adipocytes as well as beige adipocytes are preferentially derived from a Myf5-lineage because of the absence of myogenic gene expression in white adipocytes and their precursors (62, 63, 64). However, this view was recently challenged by the paradoxical overgrowth of specific WAT depots after conditional depletion of PTEN driven by Myf5-Cre (65). A subpopulation of adipocytes at some WAT depots can be derived from the Myf5+ lineage. However, Myf5+ lineage adipocytes express lower levels of thermogenic genes than Myf5-lineage adipocytes at the same depot (66).

## Plasticity of adipocytes

During the embryonic or early postnatal stage, rapidly accumulated adipocytes along with other stromal cells construct functional adipose tissues. Given that the development of an organ involves remodeling, the accumulation of adipocytes in adipose tissues necessarily involves two opposite biological processes. Differentiation generates new adipocytes, and cell death clears old or abnormal adipocytes (Fig. 1). These activities result in the renewal of adipocytes, which not only occurs during fetal development and childhood but also remains throughout adulthood. Although the number of adipocytes stays constant in adulthood in lean and obese individuals, studies analyzing the integration of ^14^C derived from nuclear bomb tests in genomic DNA and calculating the turnover rate have shown that approximately 10% of fat cells are renewed annually (67). Adipocyte turnover is relevant to the morphology and function of adipose tissue, as well as the physiological and pathological states of adipose tissues (68). For example, low adipocyte generation rates are associated with adipose tissue overgrowth without an increased number of cells (hypertrophy), whereas high generation rates are associated with adipose hyperplasia. Hypertrophic adipocytes resulting from lipid overload induce a series of adverse effects, such as hypoxia, lipotoxicity, inflammation, and metabolic impairment.

### Transcriptional regulation of adipocyte differentiation

The differentiation of adipocytes is regulated by an elaborate network involving a series of transcriptional events, in which CCAT/enhancer-binding proteins and PPARγ act as key regulators. The program of adipocyte differentiation has been extensively reviewed by many researchers during the last decade (69, 70), so we are not going to talk about this. Instead, we will discuss adipocyte death here.

### Death and senescence of adipocytes

#### Apoptosis

Most studies describe the death of adipocytes as apoptosis. Apoptosis starts with death signals coming from outside or inside the cell. The outside signals activate extrinsic/cell surface death receptors and caspase 8, whereas the inside signals activate the intrinsic pathway involving the disruption of mitochondrial membranes, release of cytochrome c from the mitochondria, and activation of caspase 9. Both the extrinsic and intrinsic pathways ultimately activate caspase 3, which causes DNA fragmentation, cytoplasmic shrinkage, and membrane blebbing. Adipocyte apoptosis is reported to be upregulated in the adipose tissues of patients with obesity and type 2 diabetes (71, 72). If mice are fed a high-fat diet, the percentage of dead epididymal adipocytes increases progressively from 0.1% at week one to as much as 16% at week 12 of high-fat feeding (73). Dead adipocytes are surrounded by macrophages that form characteristic crown-like structures associated with the production of proinflammatory cytokines (74, 75, 76). Crown-like structures have been found in adipose tissues from both obese mice and humans (73, 77, 78). Notably, adipocyte apoptosis is also present in lipoatrophic adipose tissues, which are characterized by the loss of subcutaneous adipose tissue. Long-term use of highly active antiretroviral treatment in HIV-infected patients results in the appearance of peripheral fat wasting and abdominal fat accumulation (central adiposity), the so-called HIV/highly active antiretroviral treatment–associated lipodystrophy syndrome (HALS) (79, 80). Many studies have shown adipocyte apoptosis in the lipodystrophic subcutaneous tissues of HALS patients (81, 82, 83). The association of adipocyte apoptosis with lipodystrophy suggests an important role for cell apoptosis in the progression of lipodystrophy, which is also supported by the finding that targeted activation of caspase 8 in adipocytes induces apoptosis and causes mice to acquire a lipoatrophic phenotype (84). A number of studies have ascribed adipocyte apoptosis to proinflammatory cytokines. The most important cytokine is tumor necrosis factor α (TNFα). TNFα is secreted by immune cells or hypertrophic adipocytes (85). The expression of TNFα in adipose tissue is elevated in a variety of experimental obesity models (86, 87) and obese humans (88, 89, 90), as well as in patients with HALS and cancer cachexia (91, 92). The association between a high level of TNFα and obesity/lipodystrophy suggests a role of TNFα in inducing apoptosis. *Ex vivo* experiments have shown direct evidence that TNFα induces or augments apoptosis in cultured brown and white adipocytes (93). Consistently, absence of the TNFα receptor results in a reduction in brown adipocyte apoptosis in genetically obese (ob/ob) mice (94).

#### Pyroptosis

Pyroptosis is another form of programmed cell death that is different from the condensation seen in apoptotic cells and pyroptosis features cell enlargement, and plasma membrane rupture. In pyroptotic cells, inflammatory caspase 1 is activated through the assembly of multiprotein complexes called inflammasomes. Activated caspase 1 induces pyroptosis and produces proinflammatory cytokines, such as interleukin-1β and interleukin-18 (95, 96). In leptin-deficient ob/ob mice, the formation of active caspase 1 was detected in the cytoplasm of some hypertrophic adipocytes, indicating that hypertrophic adipocytes are likely to induce obese adipocyte death *via* pyroptosis (97). Inflammation is involved in both apoptosis and pyroptosis and could be either the cause or the result of adipocyte death. Therefore, adipocyte apoptosis and pyroptosis may coincide in most situations. For example, hormone-sensitive lipase knockout along with leptin deficiency (ob/ob) reduces white fat mass, in which apoptotic and pyroptotic adipocytes coexist (98, 99).

#### Senescence

In response to a variety of stresses, mammalian cells enter into a state of persistent proliferative arrest known as cellular senescence. Senescent cells are characterized by morphological and metabolic changes and chromatin reorganization. Unlike apoptotic cells, senescent cells are viable and metabolically active and have the potential to adopt a proinflammatory phenotype featuring the secretion of soluble factors, which is collectively known as the senescence-associated secretory phenotype. Senescent cells gradually accumulate in adipose tissues with aging (100, 101). Senescent cells also accumulate in adipose tissues of mouse models and humans with diabetes and obesity (102). The link between cell senescence and obesity is further demonstrated by the finding that inducing genomic instability through ablation of polymerase η increases the number of senescent adipose tissue cells and exacerbates the adipose tissue dysfunction induced by a high-fat diet. In contrast, the prevention of cellular senescence through inhibition of the major senescence regulator p53 attenuates metabolic abnormalities (100, 102). These results indicate that reduced genome integrity plays a causative role in provoking adipocyte senescence, which leads to the development of obesity and insulin resistance. The oxidative stress, high glucose concentrations in the microenvironment, and increased insulin-like growth factor 1 and ceramide levels that are associated with obesity are thought to promote cellular senescence (103).

## Macrophages and their plasticity in adipose tissue

### Origin of macrophages

Macrophages are found in all tissues. Tissue-resident macrophages originate in different locations according to the development stage: in the extraembryonic yolk sac starting at embryonic day 7, in the fetal liver at E10.5, and in the spleen and bone marrow after postnatal formation (104, 105, 106, 107). Primitive macrophages in the yolk sac are generated without monocytic intermediates and maintain self-renewal capacity, whereas definitive hematopoiesis in the fetal liver, spleen, and bone marrow generates monocytes that are transferred to tissues through the circulation. The majority of tissues studied so far seem to have a chimeric origin of both yolk sac progenitors and circulatory monocytes, with the exception that microglia originate exclusively from yolk sac-derived progenitors (108, 109, 110). Most adult tissues maintain macrophages with minimal contribution of adult circulating monocytes. Upon tissue injury or other inflammatory challenges, monocytes enter tissues and differentiate into macrophages, which is actually in parallel with the expansion of tissue-resident macrophages (111, 112).

### Roles of macrophages in tissue development, regeneration, and remodeling

Macrophages play key roles during mammalian development and exhibit important homeostatic activities in nearly all organs (113). Remodeling deficiencies due to the absence of macrophages have been shown in several tissues, including the mammary gland, kidney, and pancreas (114, 115). The roles of macrophages in tissue development, regeneration, and remodeling are related to their versatile capacities. As phagocytes, macrophages function as scavenger cells and phagocytize the cellular debris, apoptotic cells, invading organisms, and neutrophils that are present during tissue development or following tissue injury (116, 117). Macrophages also regulate various stem cell niches. For example, a subpopulation of macrophages in the hematopoietic niche regulates the dynamics of hematopoietic stem cell release and differentiation (115, 118, 119, 120). One of the most important ways by which macrophages regulate the microenvironment is by producing growth factors and other mediators that provide trophic support to tissues. The numerous growth factors produced by macrophages include platelet-derived growth factor, insulin-like growth factor 1, and vascular endothelial growth factor α. These cytokines promote cell proliferation and blood vessel development, which together help alleviate the local hypoxia that develops following injury (121, 122, 123, 124, 125). Macrophages also produce soluble mediators such as transforming growth factor (TGF)-β1 that stimulate local (usually associated with angiogenic vessels) and recruited fibroblasts to either proliferate or differentiate into parenchymal cells (126, 127). During development or following tissue injury, the basement membrane is disrupted by macrophage-derived matrix metalloproteinases, which remodel the extracellular matrix or help facilitate the movement of inflammatory cells to the site of tissue injury or remodeling. These activities of macrophages help rebuild functional organs.

### Plasticity of adipose tissue macrophages and their relevance to adipose metabolism

**Figure 2:**
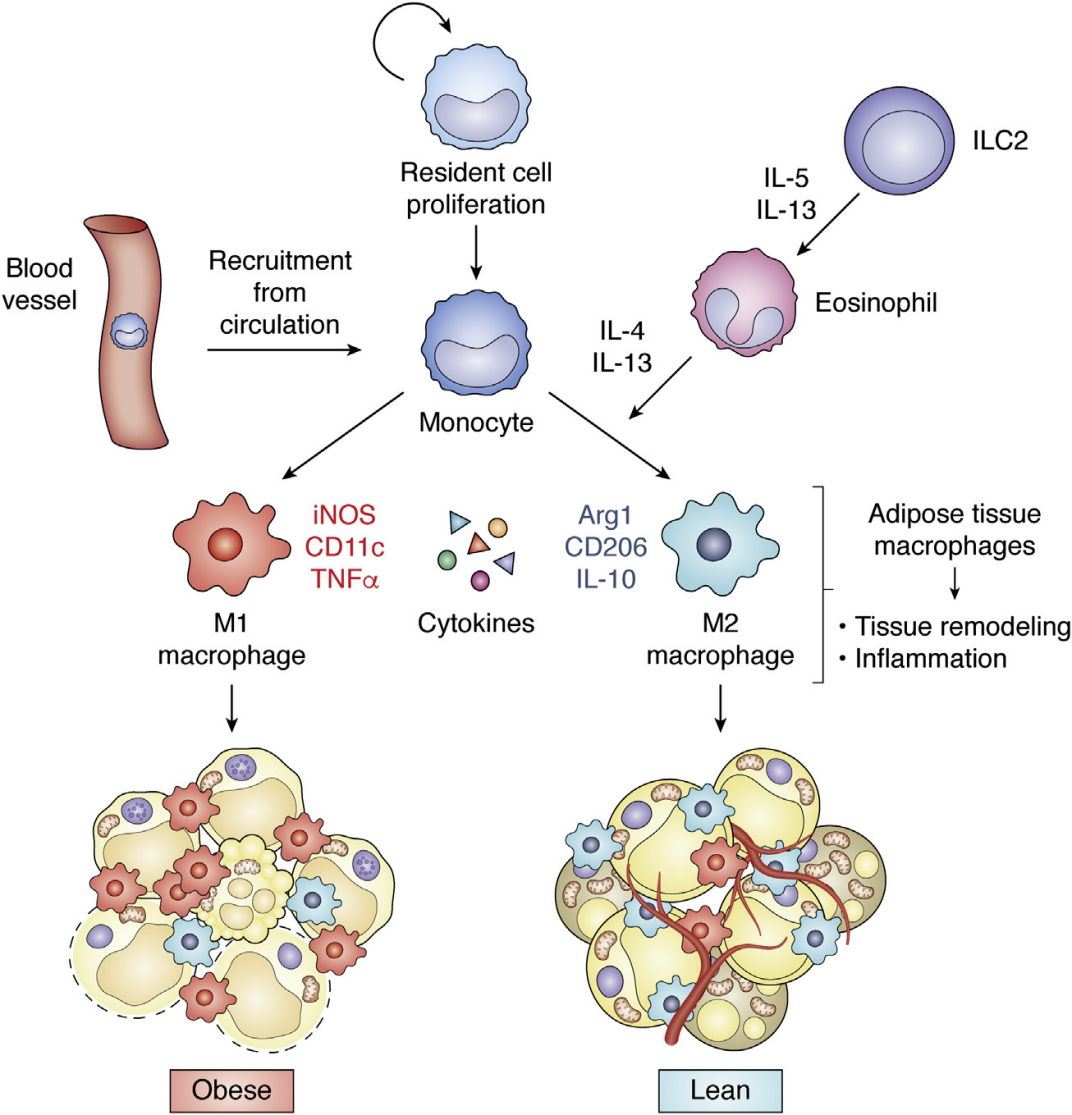
**Plasticity of adipose tissue macrophages.** Adipose tissue macrophages (ATMs) are recruited from circulation and proliferation of resident cells. ATMs play roles in tissue remodeling and inflammation. M1 macrophages catalyze arginine synthesis using nitric oxide synthase (iNOS), whereas M2 macrophages use arginase 1 (Arg1). M1 and M2 macrophages express relatively high CD11c and CD206 respectively. M1 macrophages release TNFα and favor inflammation, and are dominant in obese state, whereas M2 macrophages and their cytokine IL-10 have antiinflammatory effects and are dominant in lean state. ILC2s (type 2 innate lymphoid cells) activate and recruit eosinophils by IL-5 and IL-13. Eosinophils secrete cytokines IL-4 and IL-13 to facilitate M2 macrophage recruitment. IL, interleukin; TNFα, tumor necrosis factor α.

In addition to their trophic functions, macrophages serve as sentinel cells for the immune response, participating in tissue remodeling and inflammation (Fig. 2). Based on their functions related to inflammation, macrophages are classified as M1 (macrophages that are classically activated) or M2 (macrophages that are alternatively activated) types of cells. M1 macrophages are proinflammatory cells activated by pathogen-associated or damage-associated molecular patterns expressed by microbial pathogens (for example, lipopolysaccharide) (128, 129). M1 macrophages are characterized by the production of reactive oxygen and nitrogen species that facilitate the killing of microbial pathogens (130, 131). They also produce cytokines such as TNFα, interleukin (IL)-1β IL-12, and IL-23 to enhance the inflammatory response. M2 macrophages are thought to have antiinflammatory functions, and they are induced to polarize and proliferate by IL-4 or IL-13 through the activation of the transcription factor STAT6 (128, 132). Therefore, cells secreting IL-4 or IL-13, such as CD4+ type 2 helper T cells, basophils, eosinophils, nuocytes, and natural helper cells, contribute to M2 macrophage polarization and proliferation (133, 134, 135). M2 macrophages produce antiinflammatory cytokines such as IL-10. Accompanying these phenotypic and functional changes, the dominant metabolic pattern that macrophages rely on also alters (136). M1 macrophages rely mainly on glycolysis. In contrast, M2 cells are more dependent on oxidative phosphorylation and provide substrates for complexes of the electron transport chain, which might be controlled by PPARγ (137, 138). The metabolism of arginine is differentially regulated between the two opposite states. In M1 macrophages, arginine is catabolized to bactericidal nitric oxide and citrulline *via* the induction of inducible nitric oxide synthase, whereas M2 macrophages upregulate arginase 1, which produces the polyamine precursors urea and ornithine that are necessary for collagen synthesis and cellular proliferation, respectively (139, 140, 141).

Macrophages are highly responsive to alterations in different tissue environments (128). Considering that adipose tissues always change to metabolically adapt, adipose tissue macrophages (ATMs) should also be extremely plastic. ATMs in lean mice comprises approximately 10 to 15% of all cells in visceral fat, and obese mice show proportions up to 50% (142, 143). In humans, ATM numbers increase with obesity, from 4% in the visceral fat of lean subjects to approximately 12% in obese patients (144). The increased ATM numbers in mice and humans are partly the result of monocyte recruitment, which is mediated *via* the role of CC chemokines and their respective receptors (CCRs) (145, 146, 147). ATMs also exhibit the characters of M1 or M2 with metabolism relevance (Fig. 2). With the gain of obesity, a population of CCR2^+^ ATMs lacking galactose *N*-acetyl-galactosamine–specific lectin 1 expression (MGL1^−^) with high M1 and low M2a gene expression is recruited to and clusters around necrotic adipocytes, which express high levels of the inflammatory markers CD11c and TLR4. In contrast, M2a macrophages marked by MGL1 expression remain in interstitial spaces between adipocytes, in which IL-10 expression is prominent (148, 149). ATMs in either an M1 or M2 state are highly relevant to insulin sensitivity. Proinflammatory CD11c+ ATMs contribute to insulin resistance in human obesity (150), whereas IL-10+ ATMs preserve adipocyte insulin sensitivity in lean states (151). Notably, M2-enriched IL-10 inhibits the transcriptional elongation of TNF gene in primary macrophages (152), which is enriched in M1 cells. Therefore, ATM is more like a continuum of mixed phenotypes with either M1 or M2 being dominant. This is exemplified by the evidence that although proinflammatory M1 macrophages are dominant in the obese state, antiinflammatory macrophages are also activated, especially at the onset of high fat diet–induced obesity, in which natural killer T cells are one of the activators of M2 macrophage (153, 154, 155, 156).

In recent years, a number of studies have reported that type 2 innate lymphoid cells, eosinophils (leukocytes that respond to invading bacteria or parasites), and M2 macrophages in adipose tissue play an important role in the development of beige adipocytes. The discovery of the importance of eosinophils for browning occurred through the identification of the new signaling protein meteorin-like hormone (METRNL) (157). METRNL is released by skeletal muscles or adipose tissues upon cold stimulus and is able to activate eosinophils. Eosinophils in WAT depots were found to secrete the noninflammatory cytokines IL-4 and IL-13 to facilitate M2 macrophage recruitment (158, 159) (Fig. 2). Type 2 innate lymphoid cells were found to activate and recruit eosinophils by secreting IL-5 and IL-13, thereby leading to the alternative activation of M2 macrophages (160) (Fig. 2). One study reported that M2 macrophages synthesize and release norepinephrine (NE), which stimulate the beiging of WAT (161). However, another study argued that inhibition of tyrosine hydroxylase expression and of NE production in macrophages failed to impair the thermogenesis of adipose tissue (162). Notwithstanding the controversy regarding whether M2 macrophages release NE, the association between the recruitment of M2 macrophages and the activation of beiging shown in numerous studies indicates that other secretory factors or physical contact support from M2 macrophages might contribute to the beiging process (163, 164).

## BMP signaling participates in multiple processes involving the plasticity of adipose tissue

### BMP signaling

BMPs belong to the same TGFβ superfamily as growth/differentiation factors (GDFs), activins, nodal, and anti-Müllerian hormone (165, 166). Most ligands exhibit local paracrine activity, and some BMPs, activins, TGF-βs and GDFs are also thought to circulate and exert systemic effects (167, 168). BMPs act by binding to type I and type II BMP receptors with serine/threonine kinase activity. A mature receptor signaling complex requires one ligand dimer, two type I receptors, and two type II receptors. Ligand-binding events activate an array of downstream intracellular mediators, either the canonical SMAD pathway or p38/MAPK pathway (169, 170). Considering that there have been >30 secreted ligands, seven type I receptors, five type II receptors, and eight SMADs, gene expression activated by BMP signals is highly diverse and modified by factors such as ligand affinity and concentration, the receptor profile on the target cell, the tissue-specific transcriptional factors/cofactors, and the status of epigenetic modification (171, 172, 173, 174, 175, 176). Genes regulated by BMP ligands can therefore be variable, which enables the system to adapt to the distinct requirements for physiological or pathological activities.

### Role of BMPs in white/beige adipocyte development

**Figure 3:**
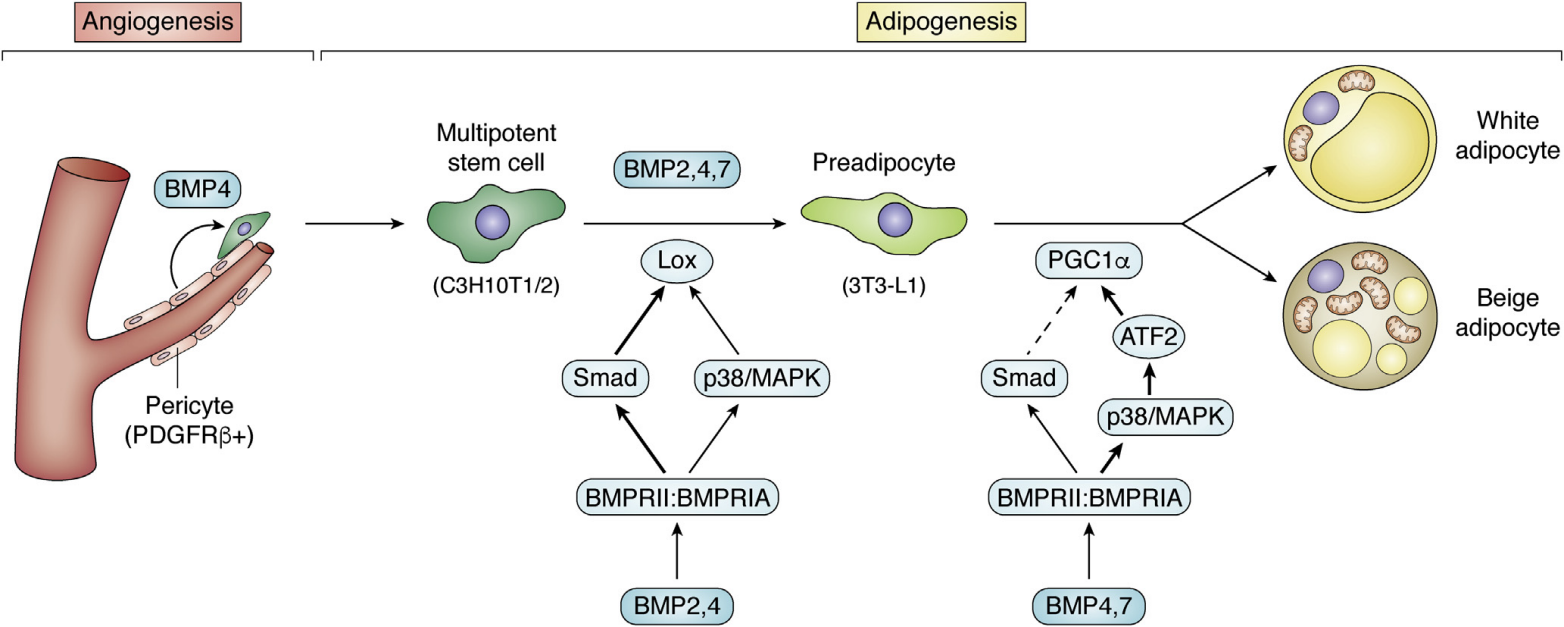
**BMPs stimulate adipogenesis at multiple stages.** BMPs are secretory proteins and expressed by both adipocyte and stromal vascular cells with higher levels in adipocytes. BMP4 stimulates angiogenesis, which serves as stem cells niches for adipogenesis; BMP2,4,7 all play roles in commitment of multipotent stem cells to preadipocytes; BMP2 and BMP4 activate Lox mainly through Smad signaling during commitment; BMP4 and BMP7 also acquires adipocyte with beige activity during terminal differentiation through P38/MAPK pathway. BMP, bone morphogenetic protein; PDGFRβ, platelet-derived growth factor receptor beta.

In the past 2 decades, studies have been carried out to understand the processes of adipocyte development. The majority of the studies were performed in cultured cell lines, among which C3H10T1/2 is the most commonly used pluripotent stem cell line; C3H10T1/2 cells can be induced to undergo adipogenic, osteogenic, or myogenic differentiation upon different treatments. Using this cell model, BMP4 was shown to have the capacity to induce adipogenic commitment (177) (Fig. 3). After treatment with recombinant BMP4, C3H10T1/2 stem cells can be induced to differentiate into adipocytes with inducers (3-isobutyl-1-methylxanthine, dexamethasone, and insulin), which only have an effect on preadipocytes. If BMP4-treated C3H10T1/2 stem cells are subcutaneously implanted into mice, they develop into adipose tissue. Further studies demonstrate that downstream Smad signaling has a dominant role in lineage determination, whereas another signaling pathway, P38/MAPK, has partial effects on commitment efficiency (178). In another study, committed preadipocytes (A33 cells) expressed BMP4 in parallel with activation of SMAD1/5/8 during proliferation. When BMP4 was blocked by Noggin, the committed proliferating A33 cells lost their preadipocyte phenotype (179). Induction of BMP4 has also been reported in primary human preadipocytes undergoing differentiation (180), further indicating a need for BMP4 for commitment.

C3H10T1/2 cells transfected with BMP2 preferentially differentiate into osteoblast-like cells with strong alkaline phosphatase–positive staining. In contrast, BMP4-transfected cells exhibit low alkaline phosphatase and mostly turn into mature adipocytes. This diversity suggests that BMP2 and BMP4 have the potential to promote the commitment of stem cells toward the osteogenic and adipogenic lineages, respectively (181). In our lab, we found that treatment with recombinant human BMP2 before administration of the adipogenic differentiation cocktail 3-isobutyl-1-methylxanthine, dexamethasone, and insulin was also able to commit C3H10T1/2 stem cells to the adipogenic lineage (Fig. 3) (182). However, there are differentially expressed genes in addition to some overlapping genes between cells treated with BMP2 and BMP4 (182). We identified a cytoskeleton-associated protein, lysyl oxidase, as one of the targets of both BMP4 and BMP2 (182). Lysyl oxidase induces an EMT-like response, which occurs and is required for the commitment process (Fig. 3). Whether the adipocytes differentiated from BMP4-committed C3H10T1/2 cells were white or brite when the model was first established was not specified (177). In a subsequent study, differentiated mature adipocytes induced by the same method was shown to express high levels of UCP-1, which was amplified by the PPARγ agonist indomethacin and/or the thyroid hormone T3 (183). Consistent with the mouse cell model, precursors from human subcutaneous adipose tissue showed higher levels of UCP-1 when BMP4 was added into the differentiation medium (184). In the C3H10T1/2 cell model, BMP7 seems to have similar effects as BMP4 on the formation of mature adipocytes, with brown-like activity indicated by the elevated expression of UCP1 and some specific markers (183). Although the author defined C3H10T1/2-derived cells as brown adipocytes, they should be different from the adipocytes developed from primary brown preadipocytes with BMP7 treatment (185).

These results obtained from *ex vivo* experiments imply that BMP4 may regulate the commitment of a subpopulation of stem cells with beige cell fate. The regulation can be expanded in *in vivo* conditions. BMP4 overexpression in adipose tissue stimulates adipogenesis at subcutaneous depots as both the fat mass and adipocyte number are increased; adipocytes within are thought to be beige cells because of their elevation of some beige-specific markers (Tbx1, Tbx15, and Hoxc9) (21). A further study showed that BMP4 commits mural cells to differentiate into preadipocytes by downregulating PDGFRβ expression (186). Moreover, BMP4 stimulates neoangiogenesis in adipose tissues (186). The angiogenic roles of BMP4 have also been documented in the generation of other tissues or during tumorigenesis (187, 188, 189). Neovascularization is important for fat expansion and beiging (190, 191, 192). In addition to supplying oxygen and nutrients to expanded fat, vascularization activates antiinflammatory M2 macrophages, which favor the beiging of adipose tissue (193, 194). Moreover, angiogenic vessels transport stem cells through circulation to local sites (57) and provide mural stem cells (38, 40, 41) for adipocyte recruitment. Collectively, BMP4 stimulates the formation of microblood vessels, which serve as stem cell niches. Meanwhile, BMP4 commits stem cells to differentiate into preadipocytes. These processes are part of the remodeling of white fat into beige fat. These results indicate that BMP4 may function at multiple stages (Fig. 3). Either BMP4 regulates the recruitment of beige cell precursors at the very beginning (186) or acquires stem cells with beige potential during commitment (183). It is also possible that BMP4 transforms white adipocytes into beige adipocytes, as PGC1α and PRDM16 are induced in adipocytes differentiated from classic white preadipocyte 3T3-L1 cells when BMP4 is present in the differentiation medium (21). Experiments crossing transgenic mice overexpressing BMP4 with lineage tracing mice may provide some evidence.

In summary, BMPs are important factors for regulating adipogenesis involving commitment, terminal differentiation, and metabolic activities. Some BMPs seem to be redundant based on the reported phenotypes. For example, BMP2, BMP4, and BMP7 individually can commit C3H10T1/2 cell to the adipogenic lineage and ultimately form adipocytes with some extent of brown activity (Fig. 3). However, whether each kind of BMP has distinguished roles needs to be determined in more *in vivo* studies, as well as examinations at the molecular level.

### Role of BMPs in brown adipocyte development

BMP7 is the BMP that is most strongly linked to brown adipogenesis. Tseng *et al.* showed that BMP7 promotes cultured brown preadipocytes to differentiate and enhances the expression of UCP1 and mitochondrial biogenesis more efficiently than any other BMP. Moreover, BMP7 knockout mice displays a marked 50% to 70% decrease in interscapular BAT mass compared with WT littermates (Table 1) (185). Lack of ALK3 (BMPRIA), which mediates BMP7 signaling, also results in impaired cervical BAT formation (Table 1). Moreover, impaired BMP signaling induces compensatory browning of white fat (195). In another study, C57BL6/J mice treated with BMP7 *via* subcutaneous osmotic minipumps for 4 weeks at 21 °C showed an increase in BAT volume (Table 1), the expression of UCP1 and hormone-sensitive lipase, and energy expenditure. These mice also displayed “browning” of WAT accompanied by diminished WAT mass (Table 1). The browning phenotype may be a direct effect of BMP7 on WAT rather than a consequence of alterations in other tissues (196).

**Table 1:** *In vivo* studies of BMPs with phenotypes related to adipose tissue

Gene/protein	Way of manipulating	Phenotype (WAT)	Phenotype (BAT)	Reference
BMP4	Ap2 promoter—transgenic	Browning of WAT WAT mass↑ Vasculature↑	Whitening of BAT	(21)
BMP4	Adenovirus injection at BAT local site	Not mentioned	Whitening of BAT	(202)
BMP4	Ap2-cre-drived knockout	WAT mass↑ Lipid accumulation↑	BAT mass↑ Lipid accumulation↑	(21)
BMP7	Whole-body null	Compensatory browning of WAT	BAT mass↓	(185)
BMP7	Injection of peptide *via* subcutaneous osmotic minipumps	Browning of WAT WAT mass↓	BAT mass↓	(196)
BMP7	Adenovirus injection *via* tail vein	No change of WAT mass	BAT mass↑	(185)
BMP8B	Whole-body knockout (challenged with HFD)	Not mentioned	BAT mass↑ Lipid droplet↑	(197)
BMP3B	Ap2 promoter—transgenic	WAT mass↓	No change of BAT mass	(224)
BMP14	Ap2 promoter—transgenic	Browning of WAT	Adipogenesis↑	(225)
BMPR1A	Myf5-cre/ap2-cre-drived knockout	Browning of WAT Innervation of WAT↑	BAT mass↓	(195)
BMPR2	Ap2-cre-drived knockout	Loss of WAT at periweaning	No change of BAT mass	(209)

BAT, brown adipose tissue; BMP, bone morphogenetic protein; WAT, white adipose tissue.

BMP8B also participates in regulating brown adipocytes but seems to be restricted to the mature adipocyte stage, which is different from BMP7. In addition to its high level in mature brown adipocytes, BMP8B is expressed in the hypothalamus, where it acts as a thermogenic protein together with AMP-activated protein kinase in key hypothalamic nuclei. Mice with BMP8B deletion exhibited altered BAT with enlarged lipid droplets and increased fat mass (Table 1), impaired thermogenesis, and susceptibility to diet-induced obesity (197, 198).

BMP6 is highly homologous with BMP7 and displays a 20-fold higher affinity for ALK3 (also known as BMP receptor IA, BMPRIA) than BMP7 and a stronger resistance to inhibition by Noggin (199). As expected, BMP6 was able to convert C2C12 murine precursor cells into preadipocytes, which were consequently induced to undergo adipogenic differentiation by a proadipogenic hormone cocktail, resulting in a bioenergetics profile similar to that of brown adipocytes (200). However, the result seems to conflict with that of the study by Tseng *et al.*, (185) which showed that in primary brown preadipocytes, BMP6 increased lipid accumulation but did not increase the expression of brown adipose genes. Another study in mature adipocytes derived from the white preadipocyte cell line 3T3-L1 showed that BMP6 increased the expression of PPARγ, which enhanced GLUT4 and insulin-mediated uptake of glucose (201). Although BMP6 shows great biochemical activity *in vitro*, the lack of *in vivo* experiments limits interest in BMP6 in metabolic research. Because BMP6 in BAT is responsive to cold (198), the undiscovered roles of BMP6 in the body deserve exploration.

Although BMP4 and BMP7 show similar effects in regulating the commitment of C3H10T1/2 cells to adipogenesis, they differentially function in BAT. To date, the reported studies support an inhibitive role of BMP4 in BAT. Overexpression of BMP4 in interscapular BAT mediated by an adenovirus containing the *bmp4* gene promoted a brown-to-white adipogenic shift by impairing the acquisition of functional thermogenesis and decreasing free fatty acid oxidation and oxygen consumption (Table 1) (202). The phenotype agrees with what was exhibited in transgenic mice with adipose-specific BMP4 overexpression in our laboratory (21), indicating that BMP4 may inhibit browning activity in BAT. Notably, BAT of BMP4-knockout (driven by the ap2 promoter) mice was also hypertrophic (Table 1), and this change was associated with markedly increased fat mass and fat droplets in brown adipocytes (21). There has been no report about the results of BMP4 knockdown meditated by local administration of adenovirus, which unanimously infects all types of cells in BAT. In fact, glucose metabolism and lipid metabolism in brown adipocytes are highly dynamic and associated with fasted and feeding states (203, 204), in which lipid loading and depletion are very obvious. Therefore, studies designed to include nutrient loading and withdrawal states may help clarify the confusing results obtained from different BMP4-manipulated mouse models.

### Role of BMPs in regulating lipid metabolism and cell survival

**Figure 4:**
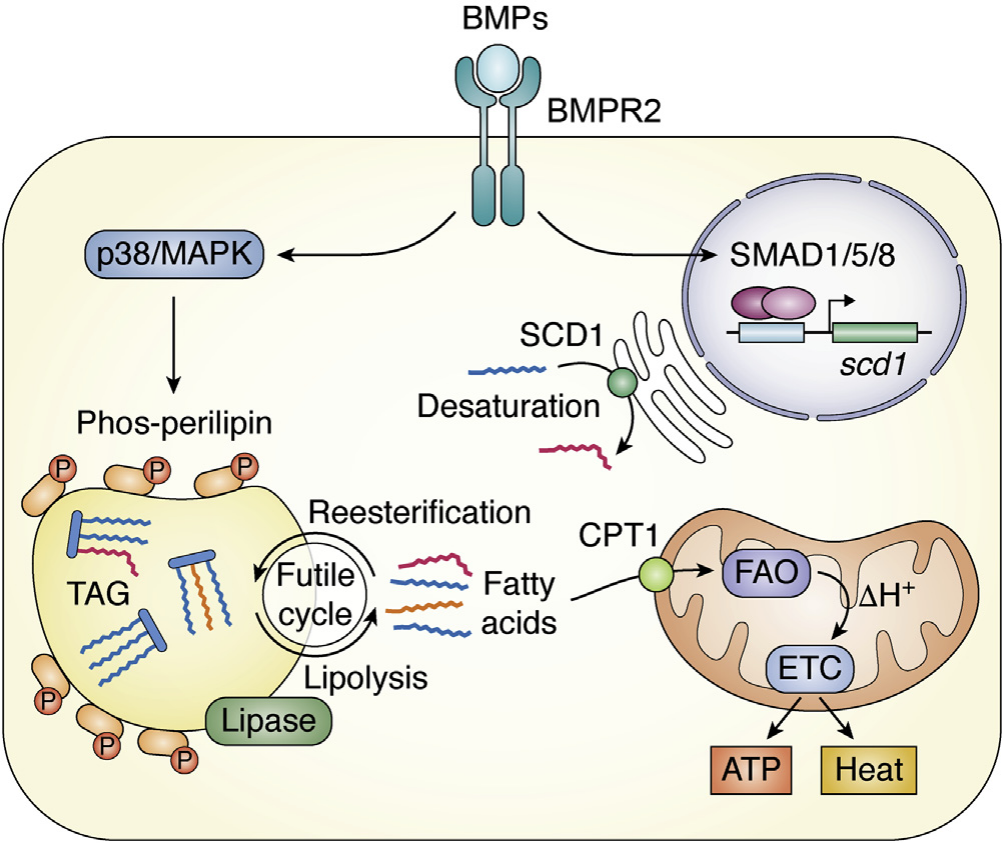
**BMPs through BMPR2 signaling stimulate catabolic metabolism of lipid.** BMPR2 activates phosphorylation of perilipin through p38/MAPK pathway to promote lipolysis. The resulting free fatty acids are transported into mitochondria and generate H^+^*via* fatty acid oxidation (FAO). H+ is transported by electron transport chain (ETC) and creates ATP or heat. A part of fatty acids is integrated into triacylglycerol (TAG) by reesterification and therefore form a futile cycle. The smad1/5/8 pathway transactivates SCD1 (stearoyl-CoA desaturase), which increased fatty acid desaturation. The resulting unsaturated fatty acids by SCD1 enzymatic activity are incorporated into the triacylglycerol, which is the necessary process of synthesis of triacylglycerol. Moreover, TAG with higher percentage of unsaturated fatty acid are more readily to mobilize. Thus, SCD1 accelerates both the synthesis and lysis of triglycerides. BMP, bone morphogenetic protein.

Although the UCP1 transcript can hardly be detected in 3T3-L1-derived adipocytes, BMP4 promotes the expression of PGC1α (21), which is key to the biogenesis of mitochondria, important organelles for fatty acid oxidation (205). The effect on mitochondria indicates that BMP4 is capable of regulating lipid metabolism. Likewise, adipocytes in WAT depots in BMP4 transgenic mice have a greater number of mitochondria than those in WT mice, along with activated fatty acid oxidation. As a result, BMP4 transgenic mice have decreased TG levels but unchanged cholesterol levels. In a cohort of 6882 human individuals, a single-nucleotide polymorphism of BMP4 was significantly related to serum TG levels but not glucose or cholesterol levels (206). A recent publication in our laboratory showed that BMP4 transcriptionally activates SCD1, an enzyme catalyzing the biosynthesis of unsaturated fatty acids. SCD1 promotes lipolysis and fat mobilization, which is fulfilled by the intermediate oleic acid (207). In fact, SCD1 accelerates both the synthesis and lysis of triglycerides, forming a somewhat futile cycle (Fig. 4) (207). We posit that this futile cycle driven by SCD1 may prime lipids in an active state ready for both incorporation and release of fatty acid and thus quickly respond to stimulation inducing energy consumption or storage, such as fasting/feeding or cold/heat exposure. BMP7 has similar function to BMP4 in increasing mitochondrial fatty acid oxidation in mature brown adipocytes; this is accompanied by an increase in fatty acid uptake and increased protein expression of CD36 and CPT1α, which import fatty acids into the mitochondria and the cell, respectively (208).

The roles of BMP4 and BMP7 in promoting lipid mobilization and fatty acid oxidation can be partly supported by evidence from a mouse model with adipose tissue–specific knockout of BMPR2, which is the common receptor for both BMP4 and BMP7. BMPR2, through p38/MAPK signaling, stimulates the phosphorylation of perilipin, which is essential for lipolysis (Fig. 4) (209). Both BMP4- and BMPR2-deficient adipocytes display a hypertrophic phenotype (21, 209). It is interesting that BMPR2 seems to have profound effects that induce obvious cell death and hypertrophy in early life (Table 1). BMPR2 knockout adipocytes are prone to pyroptosis, which leads to the release a large amount of TNFα. Notably, TNFα is able to promote lipolysis, and the resulting fatty acids might have some unrecognized roles, such as providing energy for inflammation or altering inflammatory reactions *via* lipid-regulated signaling. BMPR2-deficient adipocytes are unable to respond properly to TNFα-induced lipolysis, in part because of the impairment of perilipin phosphorylation. The reduced lipolysis and failure of fatty acid oxidation initiate mitochondrial pathway apoptosis. As a result, BMPR2 knockout adipocytes are more sensitive to death in the context of an active immune reaction, for example, in the weaning period, in which tissues undergo remarkable remodeling. These results suggest that BMPR2 is required for fat cells to effectively generate energy from stored fat for their survival in energetically demanding contexts such as inflammation.

### BMPs regulates the plasticity of macrophages

**Figure 5:**
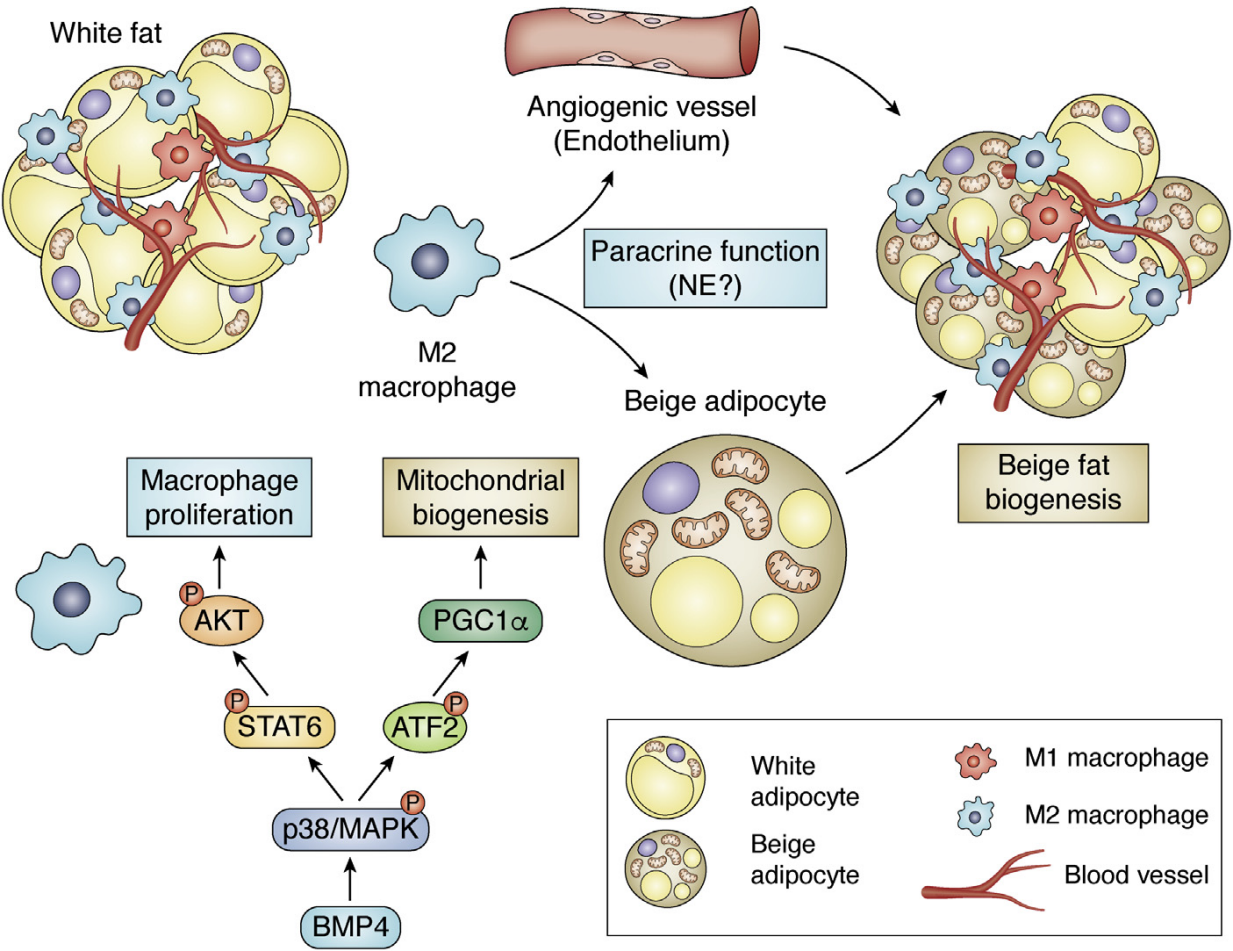
**BMP4 functions in adipocytes and macrophages to promote beige fat biogenesis.** BMP4 promotes mitochondrial biogenesis in adipocytes through P38/ATF2/PGC1α pathway; BMP4 stimulates proliferation of M2 macrophages through P38/STAT6/AKT signaling and enhances their potential for stimulating angiogenesis and beiging. M2 macrophages exert paracrine function on adipocytes and endothelia. Whether macrophage synthesize norepinephrine (NE) is controversial. BMP, bone morphogenetic protein.

We found that BMP4 alters the ATM profile in subcutaneous adipose tissue, as indicated by the data obtained in mice with genetic manipulation of BMP4 and BMPR2. In experiments in bone marrow–derived macrophages, BMP4 was demonstrated to not only activate M2 macrophages but also inhibit the activation of M1 macrophages (164). This dual function of BMP4 is consistent with the findings of another study in peritoneal macrophages (210, 211). The activation of M2 macrophages is achieved by both proliferation and the enhancement of their beige-inducing potency (Fig. 5). BMP4 stimulates proliferation of M2 macrophages through P38/STAT6/AKT signaling pathway. In addition, BMP4 potentiates M2 macrophages to induce browning-like activity of adipocytes and angiogenesis of adipose tissue (164). The role of macrophages in promoting angiogenesis may be accomplished *via* the synthesis and release of proangiogenic factors by M2 macrophages *per se* or M2 macrophage-activated mesenchymal stem cells (212).

To date, there has been no other report about the role of BMPs in regulating ATMs. However, hints can be pulled from some studies in cell models. For example, BMP7 encourages monocytes of human origin to polarize into M2 macrophages and increases the level of the antiinflammatory cytokine IL-10 (213). Therefore, BMP7 and BMP4 seem to function similarly in activating M2 polarization. In contrast, BMP6 induces the expression of inducible nitric oxide synthase and the proinflammatory factors TNF, IL1β, and IL6 in the mouse macrophage cell line RAW264.7, indicating that the macrophages are closer to the M1 type than the M2 type (214, 215, 216). The relationship between BMP2 and macrophages is mostly related to osteoclasts, which are macrophages that specifically reside in bone tissue. BMP2 markedly enhanced osteoclast differentiation and survival *via* receptor activator of nuclear factor-kappaB ligand (217, 218). BMP9 was also shown to support the function of osteoclasts (219).

### Expression of BMPs in adipose tissue and their metabolic relevance

The BMPs that have been reported to be linked to adipose tissues, and metabolic function include BMP2/4, BMP9/10, BMP5/6/7/8, and BMP3, those subgroups classified according to phylogenetic analysis. Although there may be overlap, individual BMPs preferentially enrich and function in different tissues/adipocytes.

BMP2 is expressed at higher levels in visceral than in subcutaneous WAT and in overweight and obese individuals than in lean individuals in human adipose tissues (220). Mouse studies have implicated BMP2 in white adipogenesis: ablation of Schnurri2, a gene targeted and activated by BMP2, reduced white but not brown adipose mass (221). BMP3 is also associated with overweight or obesity, as BMP3 expression in epididymal fat is substantially increased along with the development of high fat diet–induced obesity in 16-week-old mice (222). BMP3B (also referred to as GDF10) is expressed in all adipose tissues and is expressed at higher levels in preadipocytes than in mature adipocytes. Similar to BMP2 and BMP3, BMP3B increased in the mesenteric adipose tissue of mice with diet-induced obesity (223). As mesenteric adipose tissue is a representative visceral fat mainly contributing to central obesity, BMP3B might increase to counteract vicious effect caused by obesity. Therefore, it seems reasonable that transgenic mice with BMP3B overexpression in adipose tissue (directed by the aP2 promoter) appear to be protected against diet-induced obesity (224) (Table 1). BMP8B is preferentially expressed in mature brown adipocytes and white adipocytes and responds to thermogenic stimuli, including nutrient load and cold exposure. In addition to BMP8B, BMP4 and BMP6 in BAT are also increased in response to cold (198). The BMP14 (also referred to as GDF5) level is higher in interscapular BAT than in any kind of WAT in mice. Transgenic mice with adipose-specific BMP14 expression driven by the aP2 promoter have less adipose tissue and smaller adipocytes than control mice (Table 1), despite a lack of difference in food intake (225). BMP14 increases progressively in 3T3-L1 preadipocytes during adipogenic induction (226) and promotes brown adipogenesis in cells from BAT and subcutaneous WAT but not in cells from visceral WAT (225).

We found that mice highly expressed BMP4 in WAT with higher levels in the adipocyte fraction than in the stromal vascular fraction (21). Gustafson *et al.* (227) examined the BMP4 level in different populations of cells that were dissociated from human subcutaneous adipose tissue and showed that BMP4 transcript levels were high in isolated mature adipocytes. Preadipocytes also synthesize BMP4, and their mRNA and protein levels are profoundly increased after differentiation (180). Moreover, BMP4 levels in isolated mature adipocytes are positively correlated with cell size (227). Therefore, it makes sense that serum levels of BMP4 correlate positively with adipocyte diameter and negatively with insulin sensitivity (202). Considering that BMP4 has beneficial effects on energy metabolism, the correlation may represent a state of BMP4 resistance, as the elevation of BMP4 with progressing obesity is always accompanied by increased expression of BMP inhibitors, such as GREM1, which blunts BMP4’s beneficial role in metabolism (227). However, the study by Gustafson *et al.* study showed no correlation between BMP4 levels in adipocytes and BMI, which seems to conflict with the results based on our study, which showed that BMP4 expression levels in both subcutaneous and visceral WAT are negatively associated with BMI (164, 227). This paradox might have been caused by the inclusion of different types of participants. The BMI of individuals in the study by Gustafson *et al.* ranged from 19.5 to 27.5, whereas in our study, the majority of individuals were overweight (>25) and obese (>30 and up to 48.1). Because BMP4 has a beneficial effect on adipose metabolism, elevation of BMP4 is assumed to counteract the deleterious effect induced by a high-fat diet. However, if the expression of BMP4 cannot be promoted (*e.g.*, due to genetic defects), individuals might easily enter a state of obesity when the level of BMP4 is negatively associated with BMI.

The regulation of some BMPs has been shown to depend on sex. The increase in BMP8B in response to cold exposure differs between the sexes: females had 35-fold higher BAT BMP8B levels than males (198). We discovered a counter balance between BMP4 and estrogen/ERα signaling in WAT (228). Expression of BMP4 expression and its downstream signaling molecules is upregulated after menopause in female mice and human individuals. As BMP4 and ERα inhibit each other, the activation of BMP4 and its signaling may compensate for the metabolic impairment caused by lowered estrogen levels after menopause. However, if BMP4 cannot be upregulated because of genetic defects, metabolic failure related to obesity appears in aged and ovariectomized female mice, which is supported by the negative association between the BMP4 level and BMI identified in a cohort of female participants after menopause. In contrast, BMP4 defects do not result in an obese phenotype in female mice before menopause as they do for male mice because BMP4 depletion enhances ERα signaling, which displays protective effects on the metabolism of adipose tissue. These results suggest a possible reason for the discrepancy in obesity prevalence between sexes.

## Conclusion and perspective

The published data suggest that BMPs through their signaling have important roles in adipose tissue. BMPs induce various effects by regulating multiple types of cells and orchestrating them to remodel adipose tissue for adaption. For example, BMP4 stimulates angiogenesis and simultaneously commits mural cells in microvessels to adipogenesis (Fig. 3). At the same time, BMP signaling promotes mitochondrial biogenesis and lipid catabolism and acquires the recruited adipocytes with beige (Fig. 3). Moreover, BMP4 activates M2 macrophages and inhibits M1 macrophages, setting adipose tissue into a metabolically friendly immunoscenario. Activated M2 macrophages stimulate angiogenesis and beiging activity of adipocytes in a paracrine manner (Fig. 5). Taken together, the existing evidence shows that the actions of BMP4 in adipose tissue favor adipose tissue and systemic metabolism. Considering the substantial roles of BMPs in adipose tissue, genetic variations of BMPs to some extent influence the plasticity of adipose tissue and systemic metabolic phenotypes seen when individuals experience environmental changes.

To date, most studies have focused on individual BMP signaling components but have failed to consider the potential relationships between BMP types. Heterodimers formed by the combination of BMPs might influence the affinity for BMP receptors and downstream signaling. For example, BMP2 forms a heterodimer with BMP7, which promotes the phosphorylation of SMAD1/5/8 without affecting the MAPK pathway (229).

In addition to the tissue type preference of individual BMPs, the expression of BMPs also shows cell specificity within tissues. However, almost all BMPs are expressed in both mature adipocytes and the stromal vascular fraction. This fact might result from technical limitations in totally eliminating contamination from each other fraction. It is more likely that BMPs are generally expressed in many cell types. Because different types of cells communicate, precaution should be taken when making conclusions based on *ex vivo* experiments. In addition to the relationships between cells, endogenous agonists or antagonists/inhibitors may also increase the complexity of the regulatory network and often confuse outcomes from various animal models featuring gene manipulation or nutrient loading challenges. For example, the levels of BMP4 and BMP3B positively correlates with the severity of the obese phenotype, but artificial overexpression of BMP4 or BMP3B in adipocytes improves metabolism. To date, studies of BMPs in adipose tissues have seldom taken fasting and feeding states into account. As glucose metabolism and lipid metabolism in adipose tissues change profoundly, the fasting and feeding states might also affect the interpretation of the functions of BMPs.

Thus, BMPs and their signaling may serve important roles in adipose development and metabolism. A full understanding of the actions of BMP signaling would allow for the development of new strategies targeting obesity and metabolic disorders.

## Funding and additional information

The works in our lab mentioned in the article were supported by the 10.13039/100014717National Natural Science Foundation (NSFC) Grants 81730021 to Q. -Q. T. and 31670787 and 81200621 to S. Q.

## Conflict of interest

The authors declare that they have no conflicts of interest with the contents of this article.
